# Dental College Education

**Published:** 1877-01

**Authors:** H. A. Smith


### DENTAL COLLEGE EDUCATION.

BY H. A. SMITH, D. D. S.

*Read before the Ohio State Dental Society.*

A dental college is properly a technical school to prepare young men for the practice of dentistry.

Nearly forty years ago, the first institution of this kind was established, now we have eight strictly dental colleges.

These schools were created and are sustained by the dental profession, and no matter what the inherent defects of their system of instruction may be, if we exercise that charity which best begins at home, we are compelled to admit that our colleges fairly represent the present status of dentistry amongst us.

In all of these colleges, nearly the same course of instruction has been adopted, and it is but fair to presume, after an experience of these forty years, that the curriculum agreed upon has been found sufficient to qualify the student to practice the calling of a dentist.

Various and sharp criticisms have of late been made upon the methods of our dental schools. It is charged that they are not constantly refo~ming themselves, so as to adapt their course of training to the ever varying and ever advancing requirements of the age. It is quite probable, however, that the more experienced teachers in dental colleges have as accurate knowledge or as just an appreciation of the demands of the time as these self-constituted critcs, who assume the function of both assailant and judge.

In all the affairs of life there is a practical side, and in the matter of dental education there is the practicable and available method and the ideal or so called advanced system, Those who clamor for the broader culture have a mistaken notion that the badge D. D. S. means vastly more than the people who employ the dentist do.

The work which a dentist is called upon to perform is limited to a certain routine as a rule, and the public has a right to expect that he shall be able to do well, that which his title indicates he is competent to do. His degree is simply the outward sign of a standard attained. This test the public has a claim to demand, and will more and more continue to demand, just as is done in relation to medicine. For these are fields of study in which no other practical test is applicable under ordinary circumstances.

Experience has shown then that the dental curriculum is, ordinarily, full enough to qualify the dental student for his professional duties. If he lacks these qualifications, it is highly probable that he has failed to avail himself through want of capacity, or indifference of the advantages of his college course. No system can be efficient without co-operation and effort on the part of the student. In every class of dental students, we will find some who are industrious and bent on improvement, but for want of adequate preparation they are prevented receiving the full benefit of the course. At least half the time of the session is frequently lost in acquiring habits of study and observation, and in the mastery of the elementary principles of the subjects taught.

If the dental student will come to the college with this work already done, and the proposed extension of the time of the session to five or six months is carried out, there is no good reason, especially if the progressive system of instruction is adopted, why the dental graduate may not center upon the special duties of his employment as well prepared as the graduates of most of the technical schools of the country.

I have spoken of the need of pupils capable of understanding and appropriating what is taught in the dental colleges. This presupposes instructors who are qualifiied to teach. With the lecture system especially, which is generally adopted, the professor ought to have a just conception of the nature of his work. His business is to teach and to train; in order to teach he must know, and continue to study and learn. To do this he must have leisure and opportunities. For these reasons he should not be so worn down with other labors to earn a living as to have little strength left for either teaching or study. Our dental colleges being wholly without endowment funds, receive their support from lecture fees. The revenue from this source, beside being uncertain, is frequently so small that it would barely pay one efficient teacher for his time.

With the existence of this state of things, there can be no stimulus for any one to enter upon the duties and give that self-devotion to the work of teaching, without which there can be no success.

The poverty of our colleges tends to make their existence dependent upon the zeal and reputation of its faculty; frequently upon a single member of it; and if he should be called away or die the institution would have no independent life of its own.

The new movement of associating dental schools with universities or more directly with the medical department of these institutions, may be attended with some advantages. The collections, cabinets of apparatus and laboratories belonging to the universities could be utilized. Some of the professors would lecture in common to both the medical and dental classes, and thus avoid duplication of salaries and buildings. The advantage of surrounding the dental student with an atmosphere of study, such as is supposed to pervade these older institutions. It is claimed also that the tendency of this association would be to bring a superior order of young men into the dental department, fresh from having graduated in the academical department of the university. If, however, the statement that of four hundred and eleven medical students of the class of 1867–8 in Michigan University only nineteen had taken the bachelor's degree, may be accepted as an indication of what may be accomplished in this direction, it will prove, I fear, only a delusion; and that the medico-dental student will be very much after the pattern of those who compose the classes in dental colleges.

If the broader culture of the medical man is to be regarded as a *sine qua non* in the professional work of the dentist, it would appear that the only proper method, by which the student could qualify himself fully, would be to pursue his medical course to the end, without much reference to the more technical and clinical work of his specialty. "Dentistry is too broad a specialty to be taught except by those who have closely studied and practiced it, and it would be worse than absurd to attempt to make dentists and physicians at the same time." (Arthur.) The student having passed the examination for a medical degree, would enter upon a course of lectures upon the theory and practice of dentistry. In this way he will be greatly assisted in specializing his medical knowledge. This course of lectures should be supplemented by practical work in the infirmary, superintended by skillful demonstrators.

Whilst there is very much taught in a thorough general course in medicine which would be of great practical benefit to the dentist, there is also much which it is difficult to perceive would be of any use to him in practice. It is no easy business for a dentist to learn that which has a direct bearing upon his professional work; and when we reflect that most dental studeuts have to "carve out their own fortunes, and devote themselves early to the practical affairs of life," very few can give more than two or three years to the special studies of their profession. It is well therefore that a careful pruning process should be exercised, and only the more essential studies pursued. It is true of dental education as was recently said of medical education, "that in order to know a little well, we must be content to be ignorant of a great deal."

The advocates of this new departure in dental education look ultimately to the removal of the distinctive brand of the dentist. If we are agreed to do this, and admit one degree is enough, that the greater covers the less, that we can never be doctors, in the desirable fullness of the term, unless we replace the D. D. S. with the M. D., then, assuredly, the answer to the question embraced in the subject before you for discussion would be that the dental student could best pursue his studies in the medico-dental colleges. As the reform method is as yet an experiment, it remains to be seen which system will best serve the real interests of dental education.

Meanwhile let us rally to the support of our own dental schools, and make them the most efficient for the work they are intended to do. They have already accomplished much. As individual practitioners we are greatly indebted to dental colleges. It is through their agency, largely, that dentistry has attained to its present standing with the people, as a useful and distinct professional calling. All this has been affected without sympathy or recognization to any considerable extent from the medical prefession; and when I reflect upon these things I feel like clinging to the old ship which has brought us to the vantage ground already won.

